# The association between pain-related cognitive biases and their impact upon task interference by anticipating pain: a virtual reality approach

**DOI:** 10.1097/j.pain.0000000000003640

**Published:** 2025-05-13

**Authors:** Jiaojing Xu, Linda M.G. Vancleef, Erik Bongaerts, Dimitri M.L. Van Ryckeghem

**Affiliations:** a Section Experimental Health Psychology, Department of Clinical Psychological Science, Faculty of Psychology and Neuroscience, Maastricht University, Maastricht, the Netherlands; b Institute for Health and Behaviour, INSIDE, University of Luxembourg, Esch-sur-Alzette, Luxembourg; c Department of Experimental Clinical and Health Psychology, Ghent University, Ghent, Belgium

**Keywords:** Attention bias, Interpretation bias, Pain interference, Virtual reality

## Abstract

Contemporary pain theories postulate that pain-related attention bias (AB) and interpretation bias (IB) uniquely and synergistically impact upon poor pain outcomes. As yet, available research findings are scarce and mixed. The current study aimed to investigate the association between pain-related AB and IB and their impact upon task interference by impending pain. A total of 85 healthy participants performed a newly developed virtual reality (VR) paradigm allowing to simultaneously assess AB, IB, and pain-related task interference (ie, because of the presence of pain-related information) within a dynamic pain context. Particularly, participants learned to distinguish pain (CS+) from nonpain (CS−) cues, as represented by balls of varying blue shades. In a following test phase, participants completed a 2-back task while 1 of the 3 cue types (balls) was floating around in the VR environment: pain cues (CS+; light blue shades), ambiguous pain cues (CSA; mid blue shades), or nonpain cues (CS−; dark blue shades). During the entire paradigm, cue-related eye gaze (AB), pupillometry data and pain threat ratings (IB), as well as 2-back task performance (task interference) were recorded. Results indicated the presence of AB and IB for (ambiguous) pain cues. Furthermore, task interference (longer response latency) by pain-related information was observed in the presence of pain cues. In addition, results revealed a positive association between IB and AB toward identical ambiguous pain cues. Furthermore, AB and IB were found to uniquely, but not synergistically, impact upon task interference. Future validation studies replicating current findings while manipulating context features and goal pursuit are warranted.

## Abstract 2

Supplemental Digital Content is Available in the Text.

Using a virtual reality approach, current study shows that biases in attention and interpretation of pain information are interrelated and uniquely, not synergistically, predict pain interference.

## 1. Introduction

Contemporary pain theories highlight cognitive biases to be key for the development and maintenance of chronic pain.^32,43,50^ Particularly, selective attention for pain-related information, referred to as attention bias (AB)^13,44^ and biased interpretation of ambiguous information as being pain-related, referred to as interpretation bias (IB),^37^ have been extensively studied. Although AB and IB have most often been studied in isolation from each other, several researchers have argued for their simultaneous investigation to gain insight in their association as well as their unique and synergistic impact upon poor pain outcomes.^21,50,52^ So far, studies investigating the association between AB and IB revealed inconsistent findings, concerning their association^5,10,38^ as well as their impact upon pain outcomes.^21,57^

Several theoretical and methodological reasons were brought forward to elucidate existing inconsistencies in research.^5,50,57^ Theoretically, researchers have argued that cognitive biases are dynamic fluctuating phenomena, impacted by context changes, such as presence of threat, and ongoing goal pursuit.^50^ Assessing AB and IB consecutively in a different context (eg, separate paradigms) and using different pain stimuli is, therefore, expected to add variability in research findings. To reduce such variability and allow for the investigation of the role of context changes in cognitive bias dynamics and their impact upon pain outcomes, simultaneous assessment of IB and AB is key. From a methodological standpoint, inconsistency in research findings has been assigned to the use of traditional paradigms developed to research cognitive biases separately (eg, dot-probe,^44^ homograph response^37^). These paradigms typically rely on the use of symbolic representations,^5,6,56^ such as pain words^22,41^ and images,^5,11,12,42^ which have been questioned for their validity to effectively activate pain schemata.^13,44^ In addition, personal relevance of used pain information is highly variable, with large heterogeneity demonstrated in the personal relevance of pain words used in AB research.^54^ Moreover, traditional paradigms have been criticized for their low reliability (eg, dot-probe paradigm,^14^ word priming task^53^) and limited level of ecological validity.^33,49,50,57^ To overcome methodological criticisms and allow theoretical advancement in this research area, researchers have called for the development and use of more ecological valid cognitive bias paradigms.^13,44,50^

Addressing this call for methodological innovation^33,39^ (developing a virtual reality [VR] paradigm to assess AB dynamics), we developed a novel VR paradigm incorporating (ambiguous) pain-predictive cues to create a dynamic testing environment. Participants were immersed in VR while they performed a 2-back task and were exposed to conditioned pain cues (floating, blue-shaded balls) signaling potential pain upon hand contact. In doing so, this paradigm integrates the assessment of AB and IB, as well as ongoing task performance and related interference by pain-related information. We hypothesized (primary hypotheses) that participants would show increased attention for (ambiguous) pain cues compared to nonpain cues (indexing AB), higher pain threat ratings and increased pupil dilation response for ambiguous cues compared to nonpain cues (indexing IB). In addition, we hypothesized (primary hypotheses) IB, AB, and task interference to be positively associated. Finally, we explored the unique and/or synergistic impact of AB and IB upon task interference (secondary hypotheses).

## 2. Method

### 2.1. Participants

Participants were recruited through advertisements distributed around the university campus, social media, and an online recruitment platform (Sona; Sona Systems, Nijmegen, the Netherlands). Sample size was based upon a power analysis (G*power^16^) for analyses testing the primary hypotheses requiring the largest sample size (ie, bivariate correlational analyses between AB, IB, and task interference), indicating a minimum of 84 participants needed to detect at least medium effects (α = 0.05, power = 0.80). Participants were included if they were aged between 18 and 65 years, had a normal (or corrected-to-normal) vision, and good proficiency in English. Participants with current chronic pain conditions, color-blindness, the presence of an electronic implant (eg, pacemaker), past or current cardiovascular diseases, past or current psychiatric disorder (eg, depression, panic/anxiety disorder), past or current neurological disorders (eg, epilepsy), current use of recreational drug or medication, and pregnancy were excluded from participating in the current study. The study protocol was registered via OSF (https://osf.io/m3xj4) and approved by the Ethics Review Committee Psychology and Neuroscience of Maastricht University (OZL_249_20_02_2022_S1).

### 2.2. Apparatus and stimuli

#### 2.2.1. Pain stimuli

Pain stimuli consisted of electrocutaneous stimuli (ECS; bipolar; 300 Hz; 200 milliseconds; instantaneous rise and fall time) delivered through a bipolar constant current stimulator (DS5; Digitimer Ltd, Hertfordshire, United Kingdom), using 2 reusable stainless steel disk electrodes (11 mm diameter) filled with K–Y gel (Reckitt Benckiser, Slough, United Kingdom), attached to the skin next to the distal radio-ulnar articulation of the left hand. The intensity of the ECS was individually calibrated using a simple staircase procedure, starting at 0.5 mA and increasing in steps of 0.5 mA. During calibration, participants rated each stimulus on a Visual Analogue Scale ranging from 0 (“no pain”) to 100 (“unbearable pain”). Once participants reported that the stimulus reached or exceeded a rating of 80/100, the staircase procedure was stopped. The procedure was repeated 3 times, whereby the highest ECS current (measured in mA) reached during the 3 staircases was used during the Attention Bias–Interpretation Bias–Virtual Reality (ABIB-VR) paradigm.

#### 2.2.2. Apparatus and software

The VR environment was presented using a HTC VIVE pro head-mounted display (HTC Corporation, Taoyuan, Taiwan) powered by a 64-bit AMD desktop (32 GB RAM; CPU: Ryzen 9 3900 at 3.09 GHz). The VR environment was developed using Unity 2021.3.14f1 (Unity Technologies, San Francisco, CA) and presented through the Steam Source engine (Valve Corporation, Bellevue, WA).

### 2.3. Attention Bias–Interpretation Bias–Virtual Reality paradigm

**Figure 1.**
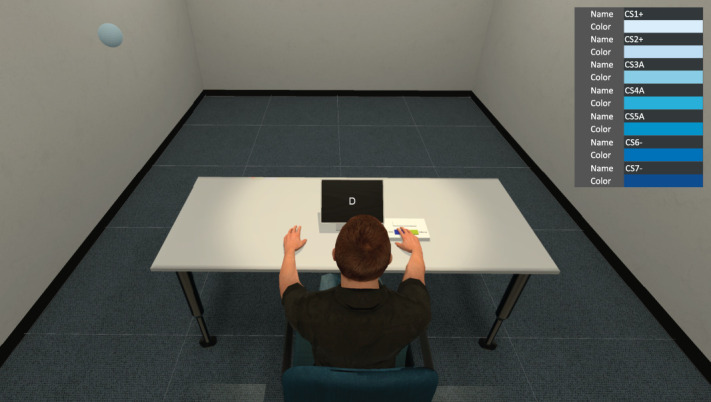
A visual representation of the test phase of the ABIB-VR paradigm, showing a floating ball (upper left) in a light blue shade. Every ball started in the upper left corner. The color gradient (upper right) of balls ranges from light blue to dark blue, consisting of 3 types of CS cues: light blue balls conditioned with pain (CS1+, CS2+), dark blue balls conditioned with no pain (CS6−, CS7−), and mid blue balls that are ambiguous in predicting pain (CS3A, CS4A, CS5A). ABIB-VR, Attention Bias–Interpretation Bias–Virtual Reality.

The ABIB-VR paradigm allows for simultaneous assessment of pain-related AB and IB as well as task interference within a dynamic pain-threat context (Fig. 1). Being immersed in VR, participants were seated in front of a tablet (30 × 20 cm) placed on a desk where they completed a 2-back task and answered Numerical Rating Scale (NRS; see below) via a response box (Cedrus-RB-844; also visualized in VR). Meanwhile, a ball (12-cm diameter) was floating around in the room (600 cm [length] × 400 cm [width] × 305 cm [height]). Crucially, this ball carried pain-related information (ie, depending upon the ball color shade, pain could be expected or not). The ABIB-VR paradigm consisted of 4 phases:

#### 2.3.1. Practice phase

During the practice phase, participants performed a 2-back task, that is, a letter memory task designed to assess working memory capacity.^28^ During this phase, a total number of 32 letters (English alphabet) were randomly displayed one by one on the tablet that was in front of the participants in the VR environment. Letters (Arial, bold, font size: 25) were displayed in white color against a black screen background. Particularly, in every letter trial, a single letter was presented in the center of the screen for 500 milliseconds, followed by a blank screen lasting for 1500 milliseconds.^7^ Participants were required to indicate whether the presented letter was the same as the one presented 2 letters earlier, using a 3-button response box positioned at the location of the right hand with the left button denoting “Same,” and the right button denoting “Different.” For example, in the sequence “T-Q-B-Q,” participants were required to respond with “Different” to the third letter “B” and with “Same” to the last letter “Q.” A next letter started if 1500 milliseconds passed. During the practice phase, participants' accuracy was recorded. Participants proceeded to the acquisition phase only when a minimum accuracy rate of 70% was reached; otherwise, the practice phase was repeated.

#### 2.3.2. Acquisition phase

**Figure 2.**
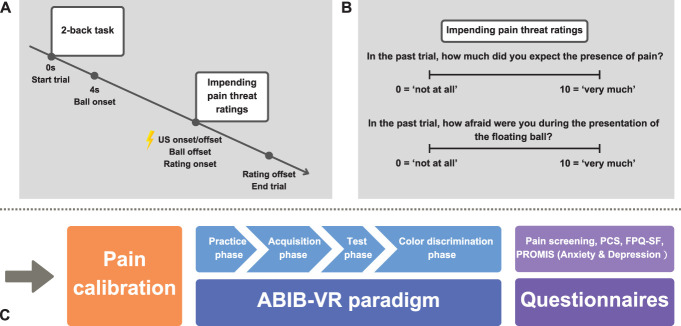
Schematic representation of the overall procedure, trial structure, and impeding pain threat ratings in each trial. (A) Trial structure in the test phase. The ball appears 4 seconds after the 2-back task began. Then, the ball was floating around in the room. Upon touching the left hand, this ball could result in the experience of a pain stimulus (CS+ in this figure, 50% reinforcement rate). Then, it disappeared and impending pain threat ratings appeared one by one. (B) Impending pain threat ratings were presented at the end of each trial, including pain expectancy (above) and pain-related fear (below), which were rated on an 11-point NRS (ranging from 0 = “not at all” to 10 = “very much”). (C) Overview of the overall procedure of the experiment. NRS, Numerical Rating Scale.

During the acquisition phase, 8 trials were presented in which a light blue (CS1+ or CS2+) or a dark blue (CS6− or CS7−; Fig. 1) ball floated around in the virtual room. Using classical conditioning, the presence of 1 of 2 blue-shaded balls (CS+; light blue) was predictive for pain (ECS; 100% reinforcement rate) at the moment participants' left hand was touched by the ball (ie, at the end of each trial; Fig. 2A). Conversely, the presence of 1 of 2 other blue-shaded balls was never linked with pain (CS−; color shades counterbalanced). To avoid predictability concerning the direction of ball movement and the timing of hand contact, 8 different ball trajectories were designed with different directions and durations (trials ranging from 16 to 58 seconds, 6-second interval). All trajectories had the same onset location (ie, upper left corner of the room) and offset location (ie, distal radio-ulnar articulation of the left hand). In this phase, 4 trajectories were randomly paired with 2 light blue balls (CS1+ or CS2+, each presented twice), whereas the remaining 4 trajectories were paired with 2 dark blue balls (CS6− or CS7−, each presented twice), resulting in a total of 8 trials. After each trial, 2 questions appeared sequentially on the tablet. These questions probed impending pain threat during the past trial, that is, pain expectancy and pain-related fear rated on an 11-point NRS (ranging from 0 = “not at all” to 10 = “very much”; Fig. 2B). Participants answered the questions using the response box, with the left and right buttons used to decrease and increase the rating on the NRS, respectively. The top button was used to confirm the answer and proceed to the next question or trial. Upon completion of the acquisition phase, participants were again asked to provide both ratings for each of the presented balls (ie, postacquisition measures).

#### 2.3.3. Test phase

The test phase combined the 2 previous phases. Specifically, participants performed a 2-back task for the entire duration of the trial, whereas a blue-shaded ball floated in the room. The onset of the ball occurred 4 seconds after the start of the 2-back task. Once the ball touched the left hand (ie, trial end), it could result in a pain stimulus (Fig. 2A). At the end of each trial, impending pain threat ratings were presented (see acquisition phase). Note that during this phase, in addition to the 4 blue-shaded balls (CS1+, CS2+, CS6−, CS7−) presented during the acquisition phase, 3 new blue-shaded balls ranging from light blue to dark blue were introduced, creating ambiguous pain cues (CS3A, CS4A, CS5A; Fig. 1). These ball colors were equidistant from the CS+ and CS− colors and were never associated with a pain stimulus when the hand was touched (see color codes in Supplementary Table S1, available at http://links.lww.com/PAIN/C284). Each ball was presented once for each of the 8 trajectories, resulting in a total of 56 test trials (see duration and number of letters for each trial/ball trajectory in Supplementary Table S2, available at http://links.lww.com/PAIN/C284). In this phase, light blue balls (CS1+, CS2+) retained their predictive value for pain to reinforce previously learned associations, yet the reinforcement rate was reduced to 50%. The test phase began with a booster phase (ie, 1 reinforced CS+ and 1 CS− trial) to ensure maintenance of previous learning. This was followed by 56 test trials presented in pseudo-randomized order. Two pain stimuli were presented every 14 trials, with a self-paced break between every block of trials. During the test phase, gaze and pupillometry data were recorded using a built-in eye-tracking equipment in the VR headset. In addition, impending pain threat ratings and 2-back task performance (response latency and button press) were registered.

#### 2.3.4. Color discrimination phase

The VR paradigm ended with a color discrimination phase designed to test whether participants were successful in discriminating between the ball colors used throughout the paradigm. In this phase, pairs of balls were presented in the center of the screen. Participants were instructed to indicate whether the displayed balls had the same or different color by pressing the left button for “Same” and the right button for “Different.” They proceeded to the next trial only after a response was registered. Responses were recorded to generate accuracy scores for each participant. Each of the 7 cues that appeared in the preceding phases was presented simultaneously with one of the other remaining cues, yielding a total of 21 trials. To avoid button-press preferences, these trials were interspersed with 21 “filler” trials, in which each cue was compared with itself 3 times, giving a total of 42 trials. An accuracy rate was calculated over the 42 comparison trials.

### 2.4. Questionnaires

The Graded Chronic Pain Scale-Revised (GCPS-R)^55^ was used to screen for the presence of chronic pain. To do so, participants filled out the first question of GCPS-R only: “In the past 3 months, how often did you have pain” with a 4-point scale (0 = “never” to 3 = “every day”).

Pain-related worrying was assessed using the 13-item Pain Catastrophizing Scale.^40^ Participants were asked to recall their past pain experience and rate the extent to which they experienced each of the 13 thoughts or feelings related to pain on a 5-point Likert scale (0 = “not at all” to 4 = “all the time”). For example, “When I am in pain, I worry all the time about whether the pain will end.” The Pain Catastrophizing Scale was found to be highly reliable.^47^ In the current study, Cronbach α was 0.89.

Fear of pain was measured via the Fear of Pain Questionnaire-Short Form,^2^ which assesses fear of pain in 4 categories: severe pain, minor pain, injection pain, and dental pain. In this study, the sum score was used as an index of fear of pain. The Fear of Pain Questionnaire-Short Form consists of 20 items rated on a 5-point Likert scale (1 = “not at all” to 5 = “extreme”), and it has demonstrated good factorial validity and reliability.^2,46^ In the current study, Cronbach α was 0.86.

Anxiety and depression were assessed using the Patient-Reported Outcomes Measurement Information System (PROMIS) Emotional Distress-Anxiety (Short Form 8a) and PROMIS Emotional Distress-Depression (Short Form 8b), respectively.^31^ Each scale measures the intended emotional state in the past week with 8 items scored on a 5-point Likert scale (1 = “never” to 5 = “always”). Raw sum scores were transformed into T-scores, where a score of 50 represents the mean with a SD of 10. Both short forms demonstrated good reliability.^19^ In the current study, Cronbach α was 0.89 for the PROMIS anxiety and 0.90 for the PROMIS depression short form.

### 2.5. Procedure

Upon arriving at the lab, participants read the information letter and signed the consent form. Afterwards, their left hand was cleaned using a commercial scrub cream, and electrodes filled with electroconductive gel (K-Y gel; Johnson & Johnson, New Brunswick, NJ) were attached. Next, the pain stimulus was calibrated, followed by an individual calibration of the VR goggles (HTC Vive Eye calibration software) at the start of the ABIB-VR paradigm. Then participants received audio-taped instructions to perform this paradigm. After completing the VR paradigm, participants provided 3 retrospective ratings on a 0 to 10 NRS (0 = “not at all” to 10 = “very much”): (1) “On average, how painful was the ECS you experienced during the ABIB-VR task” (pain intensity), (2) “On average, how fearful were you for the ECS you experienced during the ABIB-VR task” (pain-related fear), and (3) “On average, how unpleasant was the ECS you experienced during the ABIB-VR task” (pain unpleasantness). Finally, participants completed the questionnaire battery (see supra), were debriefed, and received either course credits or a €15 gift voucher (see general experimental procedure in Fig. 2C). The entire experiment lasted approximately 2 hours.

### 2.6. Data handling and preparation

During preprocessing of eye gaze and pupillometry data, a fixation was defined as gazing for at least 100 milliseconds towards the area of interest (AOI).^17,25^ Next, if eye gaze data were missing for an episode of more than 500 milliseconds, they were considered invalid data and removed from further analyses. Eye gaze data that were missing for an episode of 500 milliseconds or less (eg, eye-blinks) were reconstructed.^27^ These missing eye gaze data (eg, because of eye blinks) were replaced by the focused target before the missing data gap. For pupillometry data, a median filter (window size = 11)^20^ was first applied to smoothen the pupil size data for each eye. Margins of 100 milliseconds before and after each blink (≤500 milliseconds) were removed, and missing data during margined blinks were linearly interpolated.^9^ As recommended, the window size of the median filter, as well as the duration of the margin and blink, were determined based on empirical testing in the current study (see also Mathôt and Vilotijević^27^ adopting a 500-millisecond blink threshold). Pupil size data were then averaged across both eyes. Pupil size data of test trials without a mean baseline pupil size (baseline defined as 100 milliseconds before cue onset^26^) were excluded. Next, baseline correction^26,27^ was applied for pupil dilation response during the 2-second epoch after cue onset (2-second epoch pupil dilation response [PDR]). Finally, trials with more than 50% missing data were excluded, and participants with over 50% invalid trials were removed from the analyses.

AB indices were derived from 2 gaze measures, that is, mean dwell time and mean number of fixations, whereby the AOI consisted of the cue (floating ball), assessed during each test trial. Note that we also planned to include mean first fixation latency, but this measure was excluded from final analyses because of a confounding, that is, some participants did not show eye fixations on the ball during some of the test trials (deviation from preregistration: https://osf.io/m3xj4). Instead, we also assessed eye gaze (mean dwell time per second) towards the VR tablet (on which the 2-back task was performed) AOI, as we reasoned that the time during which participants were not looking at the tablet was time they were searching for the ball in the environment. As such, reduced dwell time on the VR tablet during CSA trials compared to dwell time during CS− trials was considered reflecting pain-related AB. Furthermore, because of the variable trial duration, these gaze measures were divided by the corresponding trial duration, resulting in 3 attention indices for each CS type (CS+, CSA, CS−): (1) dwell time per second (cue AOI), (2) number of fixations per second (cue AOI), and (3) dwell time per second (tablet AOI). To index AB for ambiguous pain cues, 2 AB indices were computed by subtracting the attention indices of CS− trials from those of CSA trials, resulting in (1) AB _Cue dwell time_ and (2) AB _Cue fixations_, whereby a positive AB index indicates that participants allocated more attention to the ambiguous pain cues compared to nonpain cues. In addition, AB _Tablet dwell time_ was included as an additional measure to reflect pain-related AB.

An IB index was derived from self-report measures assessing impending pain threat per trial. Particularly, pain threat was indexed by averaging pain expectancy and pain-related fear ratings (*r* = 0.55). To quantify IB, impending pain threat ratings were averaged per CS type (CS+, CSA, CS−). Then, the pain threat ratings of CS− trials were subtracted from those of CSA trials, resulting in an IB _Pain threat_ index, whereby a positive value indicated that participants interpreted ambiguous pain cues as more threatening compared to nonpain cues. In addition, as an exploratory index of threat, we analyzed pupillometry data. Pupil size is known to increase when being exposed to highly arousing emotional stimuli, such as pain threatening objects.^8,30^ It was expected that the presence of pain and ambiguous pain cues would increase individuals' pupil size in case of perceived threat. Therefore, 2-second epoch PDR was included as an outcome reflecting the arousal triggered by pain threat. Note that full-trial PDR was excluded because of potential confounds (deviation from preregistration: https://osf.io/m3xj4). Indeed, although the luminance of the room was controlled, the varying distance between participants' eyes and the floating balls may have changed the amount of reflected light reaching the eyes, thereby influencing pupil size.^4,24^ To quantify IB, 2-second epoch PDR were averaged for each CS type (CS+, CSA, CS−). Then, the 2-second epoch PDR of CS− trials were subtracted from those of CSA trials, resulting in an IB _PDR_ index, whereby a positive value indicated that participants interpreted ambiguous pain cues as more threatening compared to nonpain cues.

Task interference was computed from 2 task performance indices during the test phase: response latency (in millisecond) and error rate. Before the data preprocessing of response times (RTs), incorrect and late responses were removed. Furthermore, outliers, identified as deviating by 3 or more SDs from the individual mean latency, were excluded.^3,6^ Hits (for correct targets) and correct rejections (for nontargets) from each 2-back trial were used to calculate error rate (ie, 1 − [hits + correct rejections]/total trials).^7^ Next, task interference indices were calculated by subtracting 2-back task performance of CS− trials from those of CSA trials, resulting in 2 indices: (1) latency-based task interference and (2) error rate–based task interference. Positive task interference indices indicated increased levels of task interference by the presence of ambiguous pain cues.

Finally, color discrimination accuracy for each participant was calculated by dividing the number of correct responses by the total number of trials in the color discrimination task.

### 2.7. Data analyses

All data analyses were performed using SPSS (version 28.0.0.0). The significance level was set at *P* < 0.05. Statistical plots were generated using R (version 4.4.1). All data were checked for normality distribution, and nonparametric tests were used when necessary (see below).

Next, manipulation checks were performed to see if participants successfully learnt to discriminate CS+ from CS− cues using paired-samples *t* test on postacquisition pain expectancy ratings. In addition, discrimination accuracy for the blue-shaded balls was assessed. To examine the presence of IB and task interference, repeated-measures analysis of variance (rmANOVA) with CS type (CS+, CSA, CS−) as within-subject factor were performed and interpretation indices (pain threat and PDR) as well as 2-back performance indices (response latency, error rate) as outcome variables. Post hoc pairwise comparisons were conducted using paired-samples *t* tests to elucidate significant effects. Partial η^2^ was reported to quantify effect sizes.^23^ Because residuals of the gaze data analyses were not normally distributed, nonparametric analyses were used to test the effect of CS type on attention indices. Particularly, a Friedman test was used to examine the main effect of CS type. Follow-up pairwise comparisons were conducted via post hoc Wilcoxon signed-rank tests.

Next, Pearson correlations were performed to examine the associations between AB indices, IB indices, and 2-back task interference. Because IB was defined as biased pain interpretation to ambiguous pain cues, we only included AB indices, IB indices, and task interference indices toward ambiguous pain cues to keep a consistency in pain-related information. In addition to preregistered analyses examining correlations between the cognitive bias indices and task interference, we also performed a series of hierarchical regression analyses to examine whether AB and IB indices collectively contributed to 2-back task interference (latency and error rate). In light of parsimony and because the basic assumption for 2-second epoch PDR was not met (ie, increased pupil size for CS+ compared to CS− trials), IB _PDR_ was excluded from these analyses. Regression models were built following a 3-step hierarchical approach, entering Age and Sex in the first step, AB index and IB index in the second step, and the AB–IB interaction term in the final step of the regression model. All continuous predictor variables were centered before creating the interaction terms and adding them to the regression model.^29,56^ Finally, Pearson correlations were calculated to explore the interrelations between individual difference variables (ie, Pain-related worrying, Fear of pain, Depression, and Anxiety), AB, IB, and 2-back task interference (see Supplementary Table S3, available at http://links.lww.com/PAIN/C284).

## 3. Results

### 3.1. Participant and pain stimuli characteristics

A total of 92 participants participated in this study. Five participants were excluded from the final analyses because of chronic pain complaints as reported on GCPS-R,^55^ and 2 others because of computer malfunction. Therefore, the final sample consisted of 85 pain-free participants (72 women, 13 men) with a mean age of 20.99 years (SD = 5.46). Participants showed an average level of Anxiety (mean _T-score_ = 56.58; SD = 6.44) and Depression (mean _T-score_ = 51.73; SD = 6.25).^31^ The mean level of Pain-related worrying (mean = 17.27, SD = 8.55) and Fear of pain (mean = 55.04, SD = 10.66) were comparable to university student samples in previous research.^57^ The ECS used throughout the ABIB-VR paradigm, evaluated at the end of the experiment, were rated to be of moderate level of pain intensity (mean = 6.06, SD = 1.54), pain-related fear (mean = 5.42, SD = 2.20), and pain-related unpleasantness (mean = 6.41, SD = 2.23).

### 3.2. Manipulation checks

Results of the paired-samples *t* tests demonstrated a successful acquisition effect. Postacquisition pain expectancy for CS+ cues (mean = 9.12, SD = 1.38) was significantly higher than for CS− cues (mean = 0.60, SD = 1.04), *t*(84) = 40.44, *P* < 0.001, *d*_*z*_ = 4.39, indicating that participants expected more to perceive pain when being touched by a CS+ cue than by a CS− cue. In addition, participants were highly accurate during the color discrimination task (mean = 0.95, SD = 0.04), indicating that they were well able to perceptually differentiate between the 7 blue-shaded balls (cues) as used in the paradigm.

### 3.3. Attention bias, interpretation bias, and 2-back task interference

#### 3.3.1. Attention bias

Nonparametric tests were conducted to examine the effect of CS type on AB. A Friedman test indicated a significant main effect of CS type (CS+, CSA, CS−) on cue dwell time per second, χ^2^(2) = 29.17, *P* < 0.001. Post hoc Wilcoxon signed-rank tests revealed a significant higher dwell time per second for CS+ cues (mean = 2.52, SD = 8.74; *Z* = −4.19, *P* < 0.001) and CSA cues (mean = 1.78, SD = 8.32; *Z* = −5.00, *P* < 0.001) compared to CS− cues (mean = 1.25; SD = 6.71), indicating more attention was allocated to pain and ambiguous pain cues. Although pointing in the same direction, the difference in this outcome between CS+ cues and CSA cues just failed to reach significance (*Z* = −1.95, *P* = 0.05). Similarly, a Friedman test indicated a significant main effect of CS type on number of fixations per second, χ^2^(2) = 24.90, *P* < 0.001. Post hoc tests indicated significantly higher number of fixations per second for CS+ cues (mean = 1.17 × 10^−2^, SD = 3.88 × 10^−2^; *Z* = −4.38, *P* < 0.001) and CSA cues (mean = 0.81 × 10^−2^, SD = 3.70 × 10^−2^; *Z* = −3.64, *P* < 0.001) compared to CS− cues (mean = 0.63 × 10^−2^; SD = 3.13 × 10^−2^), indicating more attention was allocated toward pain and ambiguous pain cues. In addition, number of fixations per second for CS+ cues was significantly higher than for CSA cues (*Z* = −2.59, *P* = 0.01). For dwell time per second (tablet AOI), a Friedman test indicated a significant main effect of CS type, χ^2^(2) = 17.56, *P* < 0.001. Post hoc tests indicated significantly lower dwell time per second (tablet AOI) in the presence of CS+ cues (mean = 940.80, SD = 86.21; *Z* = −3.03, *P* = 0.002) and CSA cues (mean = 947.94, SD = 73.96; *Z* = −3.68, *P* < 0.001) compared to CS− cues (mean = 955.55; SD = 74.16). Although pointing in the same direction, no significant difference in this outcome was observed between the presence of CS+ cues and CSA cues (*Z* = −1.37, *P* = 0.17). These results indicated less attention was allocated to the tablet displaying 2-back task when the CS+ ball or CSA ball was floating around, which is in line with the findings including the ball AOI data.

#### 3.3.2. Interpretation bias

**Figure 3.**
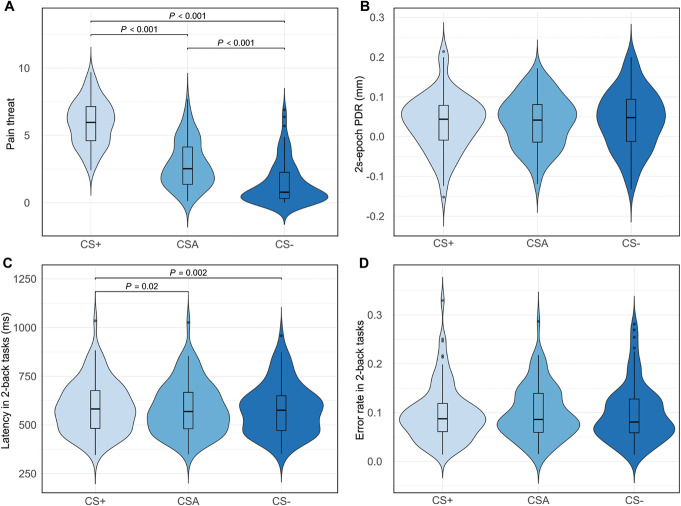
Violin-box plots of IB and task interference across different CS types. (A) Violin-box plots illustrating pain threat for each CS type. (B) Violin-box plots illustrating 2-second epoch PDR (millimeters) for each CS type. (C) Violin-box plots illustrating 2-back response latency (milliseconds) for each CS type. (D) Violin-box plots illustrating 2-back error rates for each CS type. IB, interpretation bias.

A rmANOVA with Greenhouse–Geisser correction yielded a significant effect of CS type (CS+, CSA, CS−) on pain threat, *F*(1.43,120.31) = 268.42, *P* < 0.001, partial η^2^ = 0.76. Pairwise comparisons showed a significantly higher pain threat for CS+ cues (mean = 5.92, SD = 1.68; *t*(84) = 19.01, *P* < 0.001, *d*_*z*_ = 2.06) and CSA cues (mean = 2.83, SD = 1.74; *t*(84) = 11.38, *P* < 0.001, *d*_*z*_ = 1.23) compared to CS− cues (mean = 1.44, SD = 1.58). Pain threat for CS+ cues was also significantly higher than for CSA cues (*t*(84) = 14.24, *P* < 0.001, *d*_*z*_ = 1.55; Fig. 3A). Next, a rmANOVA showed no significant effect of CS type on 2-second epoch PDR, *F*(2,168) = 0.76, *P* = 0.47, partial η^2^ = 0.01 (Fig. 3B). In sum, the self-report measure suggests that participants show an IB toward ambiguous pain cues, whereas this is not the case using the 2-second epoch PDR.

#### 3.3.3. Two-back task performance

A rmANOVA indicated a significant effect of CS type on response latency when participants performed the 2-back task, *F*(2, 168) = 6.52, *P* = 0.002, partial η^2^ = 0.07. Pair-wise comparisons indicated higher mean latency in CS+ trials (mean = 590.73, SD = 133.60) compared to CSA trials (mean = 585.27, SD = 133.14; *t*(84) = 2.29, *P* = 0.02, *d*_*z*_ = 0.25) and CS− trials (mean = 582.10, SD = 131.12; *t*(84) = 3.27, *P* =0.002, *d*_*z*_ = 0.36), indicating slowed responses in presence of pain cues (ie, pain cue interference; Fig. 3C). No difference was found between mean latency of CSA trials and CS− trials (*t*(84) = 1.43, *P* = 0.16, *d*_*z*_ = 0.16). Finally, we also performed a rmANOVA with Greenhouse–Geisser correction on task error rate. This analysis showed no significant effect of CS type on error rate, *F*(1.76, 147.63) = 0.23, *P* = 0.77, partial η^2^ = 0.003 (Fig. 3D).

### 3.4. Correlations between attention bias, interpretation bias, and 2-back task interference

Table 1 displays the correlations between AB indices, IB indices, and 2-back task interference indices. IB _Pain threat_ was significantly negatively correlated with AB _Tablet dwell time_, *r* = −0.22, *P* = 0.04, 95% confidence interval [CI] −0.42 to −0.01, indicating that participants with higher IB for ambiguous pain cues showed higher AB for these cues. No significant correlation was found between IB _Pain threat_ and the other 2 AB indices. In addition, IB _Pain threat_ was found to have a significant positive correlation with latency-based task interference, *r* = 0.22, *P* < 0.05, 95% CI 0.00-0.41, indicating that participants with higher IB showed more task interference by the presence of ambiguous pain cues. Unexpectedly, AB _Cue dwell time_ (*r* = −0.34, *P* = 0.001, 95% CI −0.52 to −0.14) and AB _Cue fixations_ (*r* = −0.38, *P* < 0.001, 95% CI −0.55 to −0.18) were found to have a significant negative correlation with latency-based task interference, indicating that participants with higher AB towards ambiguous pain cues showed less task interference by these cues. This was also aligned with the positive correlation between AB _Tablet dwell time_ (*r* = 0.23, *P* = 0.04, 95% CI 0.02-0.42) and latency-based task interference.

**Table 1:** Correlations between attention bias, interpretation bias, and 2-back task interference (N = 85).

	Mean (SD)	1	2	3	4	5	6	7
1. AB _Cue dwell time_	0.53 (1.79)	—						
2. AB _Tablet dwell time_	−7.61 (22.38)	0.07	—					
3. AB _Cue fixations_	1.84 × 10^−3^ (6.79 × 10^−3^)	0.94***	0.05	—				
4. IB _Pain threat_	1.40 (1.13)	−0.07	−0.22*	−0.19	—			
5. IB _PDR_	−3.22 × 10^−3^ (4.58 × 10^−2^)	−0.05	−0.02	−0.05	0.03	—		
6. Task interference _Error_ _rate_	2.11 × 10^−3^ (2.81 × 10^−2^)	−0.08	0.02	−0.13	0.09	−0.06	—	
7. Task interference _Latency_	3.17 (20.39)	−0.34**	0.23*	−0.38***	0.22*	0.11	−0.12	—

AB _Cue dwell time_, dwell time (per second) attention bias index for cue (ball) AOI (milliseconds per second); AB _Tablet dwell time_, dwell time (per second) attention bias index for tablet AOI (milliseconds per second); AB _Cue fixations_, number of fixations (per second) attention bias index for cue AOI; IB _Pain threat_, interpretation bias index based on 2 impending pain threat ratings; IB _PDR_, 2-second epoch PDR interpretation bias index (millimeters); Task interference _Error rate_, error rate–based task interference index; Task interference _Latency_, latency-based task interference index (milliseconds).

*P < 0.05, **P < 0.01, ***P < 0.001.

### 3.5. Simultaneous impact of attention bias and interpretation bias upon 2-back task interference

A series of stepwise hierarchical regression analyses were performed to evaluate the predictive value of AB (AB _Cue dwell time_, AB _Tablet dwell time_, AB _Cue fixations_), IB _Pain threat_, and their interaction term for 2-back task interference indices. All analyses were controlled for Age and Sex in the first step of the model (see Supplementary Tables S4.1 and S4.2, available at http://links.lww.com/PAIN/C284 for full models).

For task latency, results indicated that Age (β = 0.03, *t* = 0.32, *P* = 0.75) and Sex (β = −0.15, *t* = −1.36, *P* = 0.18) were nonsignificant predictors, *R*^2^ = 0.02, *F*(2, 82) = 0.98, *P* = 0.38. Adding AB _Cue dwell time_ (β = −0.33, *t* = −3.24, *P* = 0.002) and IB _Pain threat_ (β = 0.20, *t* = 1.92, *P* = 0.06) to the model in step 2 explained significantly more variance, Δ*R*^2^ = 0.15, Δ*F*(2, 80) = 7.50, *P* = 0.001. Adding the AB × IB interaction term (β = 0.08, *t* = 0.41, *P* = 0.69) in the third step did, however, not improve the explaining variance of the model, Δ*R*^2^ = 0.00, Δ*F*(1, 79) = 0.17, *P* = 0.69. Adding AB _Tablet dwell time_ (β = 0.29, *t* = 2.73, *P* < 0.01) and IB _Pain threat_ (β = 0.28, *t* = 2.65, *P* = 0.01) to the model in step 2 explained significantly more variance, Δ*R*^2^ = 0.13, Δ*F*(2, 80) = 5.92, *P* = 0.004. Again, adding the AB × IB interaction term (β = 0.00, *t* = −0.03, *P* = 0.98) in the third step did not improve the explained variance of the model, Δ*R*^2^ = 0.00, Δ*F*(1, 79) = 0.00, *P* = 0.98. Finally, adding AB _Cue fixations_ (β = −0.34, *t* = −3.32, *P* = 0.001) and IB _Pain threat_ (β = 0.15, *t* = 1.46, *P* = 0.15) to the model in step 2 explained significantly more variance, Δ*R*^2^ = 0.16, Δ*F*(2, 80) = 7.80, *P* < 0.001. Adding the AB × IB interaction term (β = 0.01, *t* = 0.06, *P* = 0.95) in the third step did again not improve the explaining variance, Δ*R*^2^ = 0.00, Δ*F*(1, 79) = 0.00, *P* = 0.95.

For error rate, results indicated that Age (β = 0.13, *t* = 1.20, *P* = 0.23) and Sex (β = −0.06, *t* = −0.51, *P* = 0.61) were nonsignificant predictors, *R*^2^ = 0.02, *F*(2, 82) = 0.86, *P* = 0.43. Adding IB and AB predictors (model 1: AB _Cue dwell time_, IB _Pain threat_; model 2: AB _Tablet dwell time_, IB _Pain threat_; model 3: AB _Cue fixations_, IB _Pain threat_) did not explain additional variance. However, the addition of the AB _Tablet dwell time_ × IB _Pain threat_ interaction term (β = 0.26, *t* = 2.27, *P* < 0.05) in the third step of model 2 did significantly improve the explained variance, Δ*R*^2^ = 0.06, Δ*F*(1, 79) = 5.16, *P* < 0.05. Yet, follow-up analysis including simple slopes analyses for low (−1 SD below the mean) and high values (+1 SD above the mean) of IB showed that neither of the slopes reached significance (both *t* < 1.87, ns).

## 4. Discussion

The current study set out to investigate the association between pain-related AB and IB, as well as their unique and synergistic impact upon pain-related task interference. To do so, we developed a dynamic and ecologically valid VR paradigm combining the assessment of AB, IB, and cognitive task performance. Research findings can be readily summarized. First, participants showed increased attention towards both pain and ambiguous pain information. Second, participants' interpretation of ambiguous pain information favored to be pain-related, despite never being associated with actual pain. Third, our findings indicated that 2-back task performance was slowed in the presence of (ambiguous) pain cues compared to nonpain cues. Fourth, AB and IB for ambiguous pain cues were associated. Finally, current results support a unique, but not a synergistic, contribution of AB and IB in predicting pain-related task interference. Each of current findings warrants further discussion.

Using the ABIB-VR paradigm, the current study showed that healthy people anticipating pain show biases in terms of attention and interpretation for (ambiguous) pain cues. Concerning IB, the self-report outcome was fully consistent with our hypothesis. Participants interpreted ambiguous cues, which were never associated with pain, as being more pain-related and threatening than nonpain cues. This finding adds to previous research showing that healthy participants display an IB for ambiguous stimuli when being confronted with an actual health or pain threat,^45^ and a recent study indicating that participants' interpretation of ambiguous information (ie, geometric figures carrying potential pain information) favored pain-relatedness rather than being neutral.^57^ In addition, participants' attention was drawn, that is, longer gaze duration and more fixations, on both conditioned pain cues and ambiguous pain cues. These findings corroborate with prior research indicating that healthy people anticipating pain show an AB for conditioned pain cues^35,48,58^ and have a tendency to interpret ambiguous pain cues as pain-related.^45,57^ Yet, current research extends previous findings to a more ecological valid and dynamic pain context. In the current study, participants were immersed in a VR setting, where cues predicting potential pain consists of balls floating in the environment, while performing a cognitive demanding task on a tablet. Actively searching for (ambiguous) pain-related cues mimics real-life contexts much better than passive viewing of static symbolic pain stimuli in traditional AB paradigms (eg, dot-probe).^13,50,51^ Furthermore, our data showed reduced dwell time on the tablet during CS+ and CSA trials compared to CS− trials, further evidencing that participants increasingly search for the floating cue during (ambiguous) pain trials.

Besides establishing the presence of AB and IB, we also investigated the association between AB and IB. Contrasting hypotheses, results indicated that cue-related AB was not associated with IB. Yet, the absence of this finding may be because of the use of dynamically moving (pain) cues. Indeed, people do not know where to look for the (pain) cue as it is moving throughout the room. Therefore, people may spend more time searching in the room to find the (pain) cue than actually gazing at the cue. In addition, although the use of eye tracking is often regarded as a direct assessment of attention, it does not measure peripheral vision. It is likely that during the search for the floating cue, the cue was detected in the peripheral field without actually gazing toward it.^15^ In fact, dwell time away from the tablet during CS+/CSA trials may better capture the search for dynamically moving (pain) cues. Analyses including dwell time away from the tablet during CSA vs CS− trials indicate an association between IB and AB. Particularly, it was found that AB indexed via dwell time for the tablet during CSA trials was negatively associated with IB of ambiguous pain cues. This finding suggests that people who interpret presented ambiguous cues as more pain-related are less attentive on the tablet screen (displaying the task at hand) during CSA trials compared to CS− trials. This finding can be interpreted as increased time of visually searching for the floating cue during CSA trials compared to CS− trials. The found association between AB and IB is in line with a study of Schoth et al.^38^ where a positive correlation was identified between AB and IB for identical ambiguous sensory pain words, yet contradicts some prior research using traditional paradigms, showing no significant correlation^36^ or even a negative correlation^57^ between AB and IB. It is important to note that within the current study, we used cues that were highly pain-relevant for participants as the cues could be associated with actual pain. In addition, within the current study, AB and IB were assessed at the same moment for the same dynamic stimuli, reducing noise because of context changes and different content of pain stimuli. All increasing the chance to detect an association between interpretation and attention processing.^50^ Current finding provides support for recent theories suggesting that increased pain-related IB is associated with increased AB.^43,50^ For example, the Integrated Functional-Contextual Framework on Cognitive Biases in Pain^50^ proposes that early attention is captured by ambiguous bodily sensations, which are then interpreted as either threatening or nonthreatening, which again affects later attentional processes. Within this framework, authors argue that this positive association between AB and IB is likely because of shared underlying mechanisms, that is, motivation and contextual variables—that fuel their co-occurrence. Similarly, the Threat Interpretation Model^43^ hypothesizes a relationship between pain-related interpretation and attentional biases, whereby the direction of the association between AB and IB is dependent upon threat. Here, interpreting ambiguous pain information as threatening is key for the presence of a pain-related AB. Although the current study does not allow to test the premises of both models, such as temporal pattern of cognitive biases, impact of goal pursuit, threat, and other context variables, it provides evidence for a link between AB and IB and provides a methodology to investigate their premises in future research.^57^

Finally, the current study revealed a unique, but not a synergistic, contribution of AB and IB in predicting pain-related task interference. This contrasts with the “Combined Cognitive Bias Hypothesis,” proposing that cognitive biases interact to maintain a given health problem.^18^ Particularly, results indicated that people who interpreted ambiguous pain cues to be more pain-related experienced more task interference by the presence of these ambiguous pain cues. Unexpectedly, current findings also indicated that people who showed increased AB toward pain cues (AB _Cue dwell time_, AB _Cue fixations_) and decreased AB towards the tablet during CSA trials experienced less task interference by the presence of CSA cues. Although current findings reveal that AB is associated with task interference by impending threat, the direction of this association is opposite to expectations. Some speculations can be made on why this is the case. First, it may be that reduced overt attention towards ambiguous pain cues floating in the room resulted in increased uncertainty of the location of the cue (ie, close or far from the hand), and thus increased task interference. This reasoning aligns with previous research showing uncertainty causes poor performance on tasks that were unrelated to the uncertainty.^1^ Second, it may be that participants compensated effort for showing increased attention toward ambiguous pain cues. Indeed, people may have been aware of their searching behavior and compensated by improved performance during the remainder of the task. Future research may, therefore, adopt a more challenging task or use an adaptive n-back task to increase effort needed to perform the task and thus reduce potential compensation behavior of participants.

Despite the strengths of the current study, some limitations deserve consideration. First, participants' gaze for the ball was limited. It could be that participants did not need to gaze directly at the ball to detect its location but could track the ball partly in their peripheral view during their search for the ball. Results were, however, robust and largely confirmed when analyzing the gaze pattern of the tablet. Second, using the 2-epoch PDR data, we could not establish the basic threat effects. This may be because of a focus on the 2-back task at the start of the trial (limited dwell time on the ball) or because pupil responses are more impacted by participants' cognitive effort in performing the 2-back task^4,24^ than by the threat value of the cue. Third, the 2-back task was relatively easy (low error with minimal variation), making performance (particularly error rate) susceptible to ceiling effects (see supra). Fourth, we note that the hypotheses testing for synergistic effects of AB and IB on task interference were not pre-registered, and we did not correct for multiple comparisons (see Rothman^34^ for a discussion). Therefore, a replication study building on the findings of this study is recommended. Finally, caution is needed when generalizing the findings of this study. The pain stimuli were experimentally induced, and the sample primarily consisted of female university students. Furthermore, the controlled laboratory setting limits the translation of the results to real-world scenarios. Future research could incorporate more ecologically valid scenarios involving pain-inducing activities, such as shopping in a supermarket or working on a construction site that requires movements like bending, stretching, and weightlifting.

## Conflict of interest statement

The authors have no conflicts of interest to declare.

## Supplemental digital content

Supplemental digital content associated with this article can be found online at http://links.lww.com/PAIN/C284.

## Supplementary Material

SUPPLEMENTARY MATERIAL
