# Supplementary material for: The association between pain-related cognitive biases and their impact upon task interference by anticipating pain: a virtual reality approach
Source: Pain. 2025 May 13;166(10):e468–77. doi: 10.1097/j.pain.0000000000003640 (PMC12444894; doi:10.1097/j.pain.0000000000003640)
Supplement: SUPPLEMENTARY MATERIAL [file jop-166-e468-s002.pdf]

Supplementary material

Table S1

The hex color codes for the seven blue balls in ABIB-VR paradigm

CS type	Hex color code
CS1+	DEEBF7
CS2+	C6DBEF
CS3A	9ECAE1
CS4A	6BAED6
CS5A	4292C6
CS6-	2171B5
CS7-	084594

Table S2

Trajectory duration and number of letters for each trial in ABIB-VR paradigm

	Trajectory							
	1	2	3	4	5	6	7	8
Number of letters	8	11	14	17	20	23	26	29
Duration of trajectory (s)	16	22	28	34	40	46	52	58

Notes. To increase the dynamics of the CS cues, the ball trajectories were designed to be unpredictable, with varying orientations and speeds of the balls. There are eight different trajectories in total. The duration of ball trajectory is corresponding to the trial duration.

Table S3

Correlations between individual difference variables, CBs and 2-back task interference

	AB $_{\text{Cue dwell time}}$	AB $_{\text{Tablet dwell time}}$	AB $_{\text{Cue fixations}}$	IB $_{\text{Pain threat}}$	IB $_{\text{PDR}}$	Task interference $_{\text{Latency}}$	Task interference $_{\text{Error rate}}$
PCS	.00	.10	−.03	.06	.16	−.02	−.04
FPQ-SF	.03	−.11	.02	.15	−.06	−.01	−.33**
Anxiety $_{\text{T-score}}$	−.02	.19	−.04	−.07	.18	.07	−.09
Depression $_{\text{T-score}}$	−.17	−.01	−.11	−.09	.09	.03	.12

Notes. PCS: Pain Catastrophizing Scale. FPQ-SF: The Fear of Pain Questionnaire - Short Form. AB $_{\text{Cue dwell time}}$: dwell time (per second) attention bias index for cue (ball) AOI (ms per second). AB $_{\text{Tablet dwell time}}$: dwell time (per second) attention bias index for tablet AOI (ms per second). AB $_{\text{Cue fixations}}$: number of fixations (per second) attention bias index for cue AOI. IB $_{\text{Pain threat}}$: interpretation bias index based on two impending pain threat ratings. IB $_{\text{PDR}}$: 2s-epoch PDR interpretation bias index (mm). Task interference $_{\text{Latency}}$: latency-based task interference index (ms).Task interference $_{\text{Error rate}}$: error-rate-based task interference index (not percentage score).

$^{**}P < .01$

Table S4.1

Predictive values of AB and self-report IB toward latency-based 2-back task interference (N = 85)

Model	Criterion variable	Step	Predictor	β	F	R^2	ΔR^2
1. AB $_{Cue\ dwell\ time}$ - IB $_{Pain\ threat}$	Task interference $_{Latency}$	1	Age	.03	.98	.02	
			Gender	−.15			
		2	Age	.00	4.32**	.18	.15
			Gender	−.15			
			AB	−.33*			
			IB	.20			
		3	Age	.00	3.45**	.18	.00
			Gender	−.15			
			AB	−.26			
			IB	.22			
			AB × IB	.08			
2. AB $_{Tablet\ dwell\ time}$ - IB $_{Pain\ threat}$		2	Age	.01	3.51*	.15	.13
			Gender	−.15			
			AB	.29**			
			IB	.28**			
		3	Age	.01	2.77*	.15	.00
			Gender	−.15			
			AB	.29**			
			IB	.28*			
			AB × IB	.00			
3. AB $_{Cue\ fixations}$ - IB $_{Pain\ threat}$		2	Age	.00	4.47**	.18	.16
			Gender	−.13			
			AB	−.34**			
			IB	.15			
		3	Age	.00	3.53**	.18	.00
			Gender	−.13			
			AB	−.34**			
			IB	.15			
			AB × IB	.01			

Notes. AB $_{Cue\ dwell\ time}$: dwell time (per second) attention bias index for cue (ball) AOI (ms per second). AB $_{Tablet\ dwell\ time}$: dwell time (per second) attention bias index for tablet AOI (ms per second). AB $_{Cue\ fixations}$: number of fixations (per second) attention bias index for cue AOI. IB $_{Pain\ threat}$: interpretation bias index based on two impending pain threat ratings. Task interference $_{Latency}$: latency-based task interference index (ms).

$^{*}P < .05$, $^{**}P < .01$, $^{***}P < .001$.

Table S4.2

Predictive values of AB and self-report IB toward error-rate-based 2-back task interference (N = 85)

Model	Criterion variable	Step	Predictor	β	F	R^2	ΔR^2
1. AB $_{\text{Cue dwell time}}$ - IB $_{\text{Pain threat}}$	Task interference $_{\text{Error rate}}$	1	Age	.13	.86	.02	
			Gender	−.06			
		2	Age	.12	.66	.03	.01
			Gender	−.06			
			AB	−.07			
			IB	.08			
		3	Age	.13	.55	.03	.00
			Gender	−.06			
			AB	−.01			
			IB	.10			
			AB × IB	.07			
2. AB $_{\text{Tablet dwell time}}$ - IB $_{\text{Pain threat}}$		2	Age	.13	.60	.03	.01
			Gender	−.06			
			AB	.03			
			IB	.09			
		3	Age	.11	1.53	.09	.06
			Gender	.00			
			AB	.08			
			IB	.12			
			AB × IB	.26*			
3. AB $_{\text{Cue fixations}}$ - IB $_{\text{Pain threat}}$		2	Age	.12	.77	.04	.02
			Gender	−.05			
			AB	−.10			
			IB	.07			
		3	Age	.12	.62	.04	.00
			Gender	−.06			
			AB	−.09			
			IB	.08			
			AB × IB	.03			

Notes. AB $_{\text{Cue dwell time}}$: dwell time (per second) attention bias index for cue (ball) AOI (ms per second). AB $_{\text{Tablet dwell time}}$: dwell time (per second) attention bias index for tablet AOI (ms per second). AB $_{\text{Cue fixations}}$: number of fixations (per second) attention bias index for cue AOI. IB $_{\text{Pain threat}}$: interpretation bias index based on two impending pain threat ratings. Task interference $_{\text{Error rate}}$: error-rate-based task interference index (not percentage score).

*P < .05, **P < .01, ***P < .001.
